# Telomere Dysfunction in Oocytes and Embryos From Obese Mice

**DOI:** 10.3389/fcell.2021.617225

**Published:** 2021-01-21

**Authors:** Juan Ge, Congyang Li, Hongzheng Sun, Yongan Xin, Shuai Zhu, Yuan Liu, Shoubin Tang, Longsen Han, Zhenyue Huang, Qiang Wang

**Affiliations:** ^1^State Key Laboratory of Reproductive Medicine, Suzhou Municipal Hospital, Nanjing Medical University, Nanjing, China; ^2^College of Animal Science and Technology, Nanjing Agricultural University, Nanjing, China; ^3^Center for Global Health, School of Public Health, Nanjing Medical University, Nanjing, China

**Keywords:** obesity, oocyte, embryo, telomere, reproduction

## Abstract

Maternal obesity impairs oocyte quality and embryo development. However, the potential molecular pathways remain to be explored. In the present study, we examined the effects of obesity on telomere status in oocytes and embryos obtained from mice fed with high-fat diet (HFD). Of note, telomere shortening was observed in both oocytes and early embryos from obese mice, as evidenced by the reduced expression of telomerase reverse transcriptase and activity of telomerase. Moreover, quantitative analysis of telomere dysfunction-induced foci (TIFs) revealed that maternal obesity induces the defective telomeres in oocytes and embryos. Meanwhile, the high frequency of aneuploidy was detected in HFD oocytes and embryos as compared to controls, accompanying with the increased incidence of apoptotic blastocysts. In conclusion, these results indicate that telomere dysfunction might be a molecular pathway mediating the effects of maternal obesity on oocyte quality and embryo development.

## Introduction

Obesity is a common health concern in recent decades, with the prevalence of reproductive troubles among obese women increasing rapidly. Substantial studies have demonstrated that obesity disturbs multiple processes relevant to female reproduction, such as gonadotropin level, conception rate, fetal growth, as well as neonatal development (Devlieger et al., 2008; Robker, 2008). Overweight women in ovulating regularly is still accompanied with reduced pregnancy rates, indicating that obesity may interfere with the critical events of conception, specifically, oocyte/embryo quality (van der Steeg et al., 2008; Licea-Cejudo et al., 2020). Clinical data from oocyte donation and embryo transfer also suggest that poor oocyte quality contributes to the pregnancy complications experienced by obese women (Luke et al., 2011; Luzzo et al., 2012).

Telomeres consist of telomeric DNA (TTAGGG) and related proteins located at the end of eukaryotic chromosomes (Saikhun et al., 2004). They play a crucial role in stabilizing the chromosomes and preventing illegitimate chromosomal recombination (Zhao et al., 2014; Ge et al., 2019). Telomerase is a ribonucleoprotein that elongates telomeric DNA sequences, participating in the maintenance of telomere length by compensating for the loss of telomeric DNA in cell divisions (Herrera et al., 1999). Telomere length, as a significant biological marker, affects diverse cellular events such as meiosis, genomic integrity, and nuclear organization throughout the lifespan of eukaryotic cells (Noguera et al., 2020). Obesity is known to reduce telomere length via influencing systemic inflammation and redox homeostasis continuously (Mundstock et al., 2015). It has been widely reported that maternal pre-pregnancy body mass index (BMI) is associated with shorter telomere lengths in both newborn and older children (Martens et al., 2016; Clemente et al., 2019; McAninch et al., 2020). It is worth noting that exposure to maternal obesity causes poor oocyte quality and developmental defects of embryos. However, much remains to be investigated about the potential mechanisms mediating this process. In the present study, we particularly focus on the telomeric status in oocytes and preimplantation embryos from obese mice, and report our findings below.

## Results

### Reduced Telomere Length in Oocytes and Embryos From Obese Mice

**FIGURE 1:**
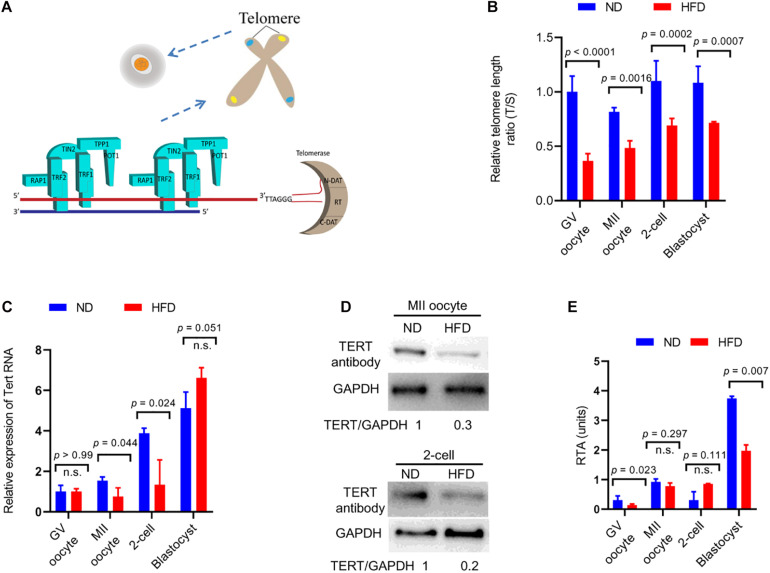
Reduced telomere length in oocytes and embryos from obese mice. **(A)** Schematic diagram showing telomere structure, telomere-associated proteins, and telomerase in cells. Telomere and telomere-associated proteins include TRF1 (telomeric repeat binding factor 1), TRF2 (telomeric repeat binding factor 2), POT1 (protection of telomeres 1), TIN2 (TRF1-interacting nuclear protein-2), TPP1 (TIN2 interacting protein), and RAP1 (repressor-activator protein 1). Telomerase are composed of N-DAT (N-terminal domain), RT (DNA- and RNA-binding regions, a central catalytic reverse transcriptase domain), C-DAT (C-terminal domain). **(B)** Relative telomere length is expressed as a T/S ratio by quantitative real-time PCR analysis (*n* = 50 oocytes/embryos from 3 mice for each group). **(C)** The relative mRNA levels of Tert in GV/MII oocytes and 2-cell/blastocysts from ND and HFD mice were detected using qRT-PCR (*n* = 50 oocytes/embryos from 3 mice for each group). **(D)** The protein levels of TERT in MII oocytes and 2-cell embryos from ND and HFD mice were evaluated by Western Blot (*n* = 200 oocytes/embryos from 10 mice for each lane). GAPDH served as an internal control. **(E)** Relative telomerase activity (RTA) in GV/MII oocytes and 2-cell/blastocysts from ND and HFD mice was measured (*n* = 50 oocytes/embryos from 3 mice for each group). Data are expressed as mean percentage ± SD, of three independent experiments. A Student’s *t*-test was used for statistical analysis; n.s., not significant.

Four-week old female mice were fed either a high-fat diet (HFD) or a normal diet (ND) for 16 weeks, and their oocytes/embryos were collected for the assessment of telomeric status, respectively. Telomeres are specialized repetitive sequences at the end of eukaryotic chromosomes, composed of non-coding DNA and a variety of proteins (Figure 1A). Here we first quantified the telomere length by telomere to single-copy gene ratio (T/S) as described previously (Callicott and Womack, 2006). Remarkably, the telomere length was decreased by ∼50% in GV/MII oocytes from HFD mice compared with ND oocytes. Likewise, ∼30% reduction in telomere length was detected in HFD 2-cell/blastocyst embryos relative to ND embryos (Figure 1B). Telomerase reverse transcriptase (TERT) is the rate-limiting catalytic subunit of telomerase, an RNA-dependent DNA polymerase that lengthens telomeric DNA to maintain telomere homeostasis (Patel et al., 2020). In line with this notion, we noticed that both mRNA and protein levels of TERT in MII oocytes and 2-cell embryos from HFD mice were reduced in comparison to controls (Figures 1C,D). However, TERT expression has no significant difference in GV oocytes and blastocyst embryos between ND and HFD groups. Therefore, we further evaluated the relative telomerase activity (RTA) in these cells using quantitative telomerase repeat amplification protocol. Interestingly, RTA levels in GV oocytes and blastocyst embryos obtained from HFD mice were significantly lower than that in ND cells (Figure 1E), indicating that both telomerase expression and activity involve in the control of telomere length. Altogether, the results suggest that maternal obesity induces a decline in telomere length in oocytes and early embryos.

### Telomere Dysfunction and DNA Damage in HFD Oocytes and Embryos

**FIGURE 2:**
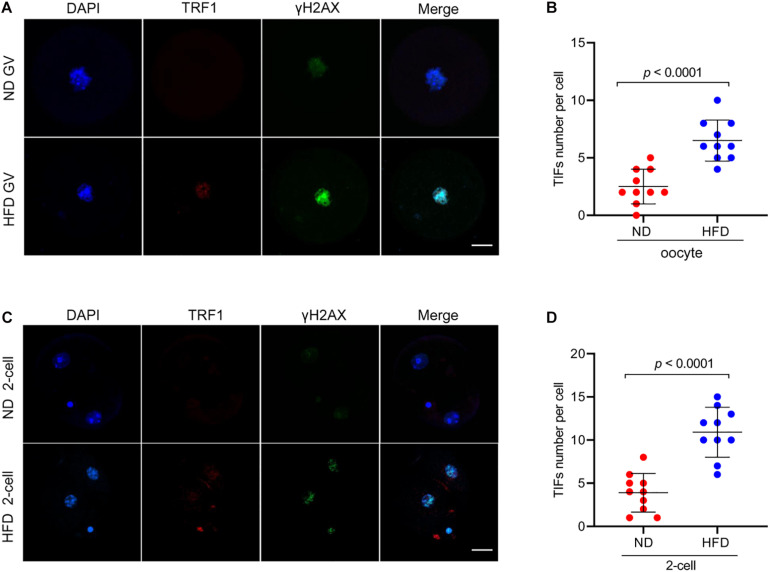
Telomere dysfunction and DNA damage in HFD oocytes and embryos. **(A)** Representative images of ND and HFD GV oocytes stained with antibodies against TRF1 (red) and γH2AX (green), and co-stained with Hoechst 33342 for chromosomes (blue). **(B)** Quantification of the numbers of TRF1 and γ-H2AX foci (TIFs) in GV oocytes. TIFs were detected by co-localization of TRF1 and γ-H2AX. Each data point represents one oocyte (*n* = 10 oocytes for each group). **(C)** Representative images of ND and HFD 2-cell embryos stained with antibodies against TRF1 (red) and γH2AX (green), and co-stained with Hoechst 33342 for chromosomes (blue). **(D)** Quantification of the numbers of TRF1 and γ-H2AX foci (TIFs) in 2-cell embryos. Each data point represents an embryo (*n* = 10 embryos for each group). Scale bars, 25 μm. Data are presented as means ± SD, a Student’s *t*-test was used for statistical analysis.

Telomere repeat-binding factor 1 (TRF1), as a telomeric double-stranded DNA binding protein, negatively regulates telomerase-dependent elongation via blocking telomerase access to the telomeres (Azzalin et al., 2007; Okamoto et al., 2008). γH2AX is an early cellular response to the induction of DNA double-strand breaks (DSBs) (Ibuki et al., 2015). Telomere dysfunction could give rise to phosphorylation of histone H2AX and TRF1 accumulation through the activation of DNA damage response factor (Fernando et al., 2011). To check the telomere function, oocytes and 2-cell embryos derived from *in vitro* fertilization (IVF) were immunolabeled with γH2AX and TRF1 antibodies simultaneously. Remarkably, as shown in Figures 2A–D, HFD oocytes and embryos accumulate more TIFs spots as compared to normal cells. These data indicate that maternal obesity is capable of inducing DNA lesions and telomere dysfunction in oocytes and preimplantation embryos.

### Genomic Instability in Oocytes and Embryos From Obese Mice

**FIGURE 3:**
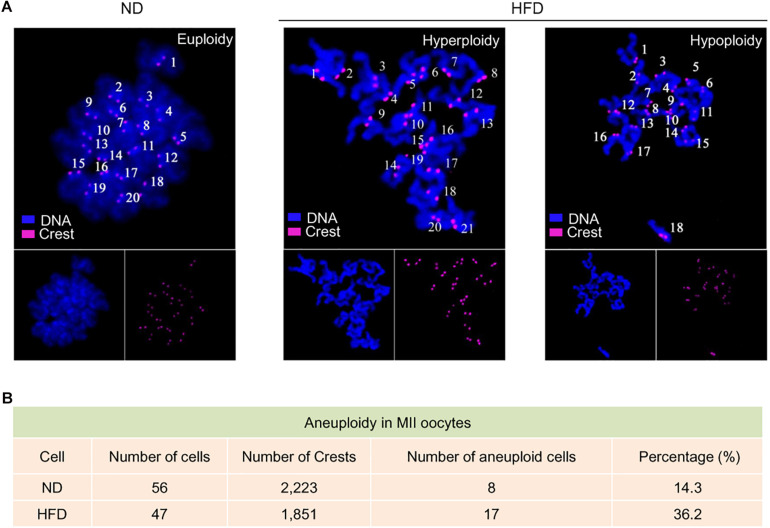
Genomic instability in oocytes from obese mice. **(A)** Chromosome spread of MII oocytes obtained from ND and HFD mice (*n* = 56 ND oocytes from 3 mice and *n* = 47 HFD oocytes from 4 mice). Purple, kinetochores stained with CREST antibody; Blue, chromosomes stained with DAPI. Representative confocal images show the euploidy in ND oocytes and aneuploidy in HFD oocytes. **(B)** Summary of the frequency of aneuploidy in ND and HFD oocytes.

**FIGURE 4:**
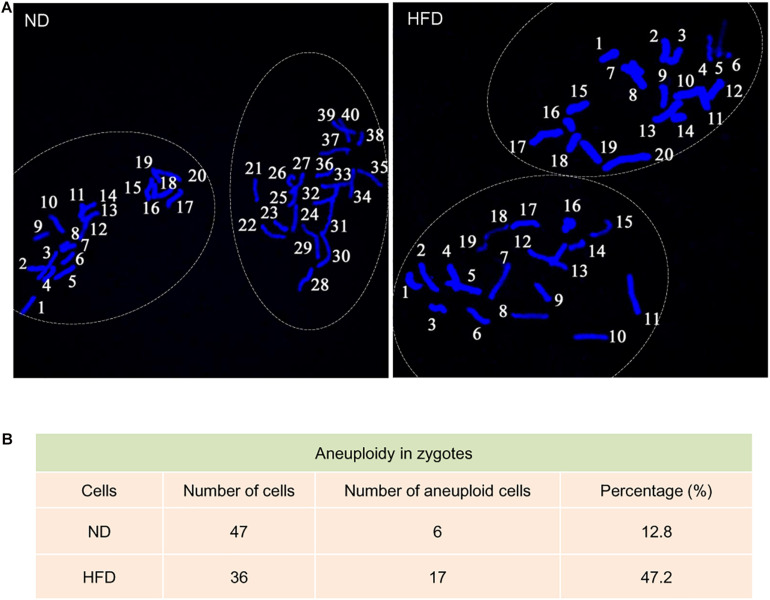
Increased aneuploidy in zygotes derived from HFD oocytes. **(A)** Chromosome spread of zygotes derived from ND and HFD oocytes (*n* = 47 ND zygotes from 5 mice and *n* = 36 HFD zygotes from 5 mice). Blue, chromosomes stained with DAPI. Representative confocal images show the euploidy in ND zygotes and aneuploidy in HFD zygotes. **(B)** Summary of the frequency of aneuploidy in ND and HFD zygotes.

Telomeres, as a special heterochromatic structure at the ends of linear chromosomes, can be utilized to prevent end-to-end fusion, nucleolytic degradation and irregular recombination (Meena et al., 2015). Hence, telomere shortening might lead to chromosomal instability in cells (Mania et al., 2014). To evaluate the genomic stability in HFD oocytes, karyotype analysis was conducted via chromosome spreading in combination with kinetochore labeling. About 3-fold increase in aneuploidy incidence in HFD oocytes was detected compared to ND oocytes (Figure 3A). Furthermore, we postulated that chromosomal abnormalities in HFD oocytes would also result in the generation of aneuploid embryos. Consistent with this hypothesis, the proportion of aneuploidy was dramatically elevated in zygotes derived from HFD oocytes (Figures 4A,B). Together, these observations indicate the genomic instability in oocytes and embryos exposed to maternal obesity, which is likely associated with the telomere dysfunction.

### Cell Apoptosis of Blastocysts Derived From Obese Mice

**FIGURE 5:**
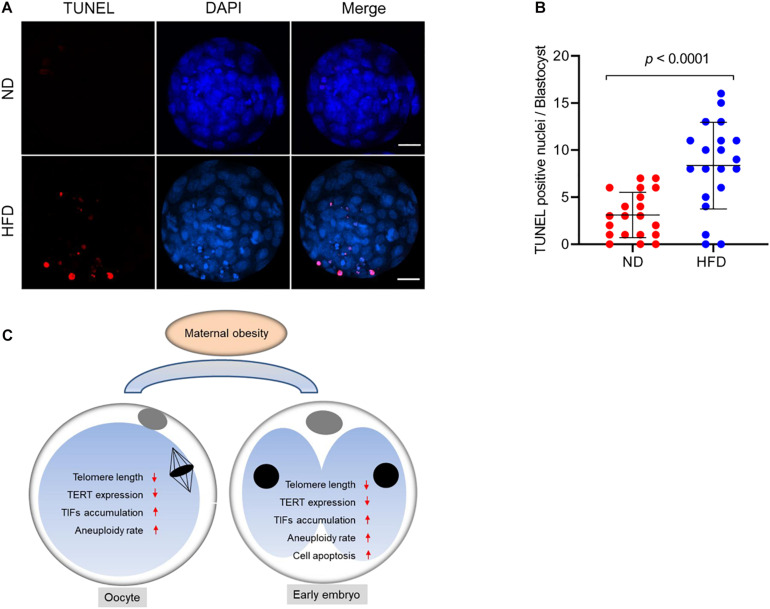
Cell apoptosis of blastocysts derived from HFD oocytes. **(A)** TUNEL analysis of blastocysts originated from ND and HFD oocytes. Embryos were labeled with Hoechst 33342 (blue) for DNA and by TUNEL for fragmented DNA (red). Scale bars, 25 μm. **(B)** Quantification of ND and HFD blastocysts apoptosis with TUNEL positive nuclei (*n* = 20 embryos from 5 mice for each group). Data are expressed as mean percentage ± SD, a Student’s *t*-test was used for statistical analysis. **(C)** Diagram summarizing the main effects of maternal obesity on telomeric status and genomic stability in oocytes and early embryos.

In mitotic cells, telomere dysfunction triggers cellular stress, perhaps via DNA damage, resulting in growth retardation and apoptosis (Harrington and Robinson, 2002). Likewise, a surveillance mechanism in germ cells has been identified to specifically target cells with dysfunctional telomeres for apoptosis (Hemann et al., 2001). Considering the shorten telomere and accumulated DNA damage in HFD oocytes, we decided to assess the apoptotic status in the resultant embryos. By performing Terminal dUTP Nick End Labeling (TUNEL) analysis, we found that TUNEL positive nuclei were hardly observed in normal blastocyst. By contrast, the apoptotic blastocysts were readily detected in obese mice (Figures 5A,B). These findings imply that defective telomere might be a factor contributing to the compromised developmental potential of HFD embryos.

## Discussion

Maternal obesity in humans alters metabolic adjustments and affects reproductive capacity including perinatal complications, preterm delivery and neonatal conditions (Bolumar et al., 2000; Wang et al., 2002). In particular, we found the impaired developmental potential of preimplantation embryos and fetal growth retardation in HFD mice (Han et al., 2018). Recently, emerging data derived from embryo transfer experiment suggests that these developmental defects are probably due to the poor oocyte quality (Wyman et al., 2008; Huypens et al., 2016).

Telomere is a protective nucleoprotein structure at the end of chromosomes, which can determine if it is the natural chromosomal termini and avoid the induction of double-strand breaks in DNA (Okamoto et al., 2008). On the other hand, telomeres prevent incomplete replication from deterioration or from fusion with chromosome ends and participate in genome regulation and protein coding during gametogenesis, especially in meiosis (Reig-Viader et al., 2016). In the present study, the reduction in telomere length in oocytes and early embryos from HFD mice was identified (Figure 1). Telomerase consists of two major components: a reverse transcriptase enzyme (TERT) and a long non-coding RNA. The telomerase activity is significantly active in germ cells and early embryos to ensure that telomere length can be restored for next generation (Schaetzlein et al., 2004). In support of this conception, both Tert expression and telomerase activity were discovered to be altered in HFD oocytes and embryos (Figure 1). Consequently, telomere homeostasis is disrupted in germline, with the production of aneuploid gametes and deficient gametogenesis, inducing fertility problems. Notably, by spreading chromosomes, we revealed the significantly increased incidence of aneuploidy in both HFD oocytes and preimplantation embryos (Figures 3, 4). The survival rate of embryos is restricted by many factors, the most prominent of which is chromosome state (Gianaroli et al., 2010). In addition, telomeres play important functions in many aspects during gametogenesis and embryogenesis, such as chromosome orientation, synapses and separation. Length of oocyte telomeres is closely related to embryonic development, and thereupon females fertility (Turner and Hartshorne, 2013). Cytogenetic abnormalities in germline can be transmitted to next generation and is catastrophic for the development of future generation. Collectively, it is conceivable that telomere dysfunction might be one of reasons for miscarriage and reproductive failure experienced by obese women.

Programmed cell death is not only essential for gametogenesis and embryogenesis, but also plays a role in the formation of fetal organs and structures. The chromosome ends are relaxed during genome replication, and the structure of telomeres is dynamic intrinsically. In order to accomplish replications, telomeres may switch between protected and deprotected states during cell cycle. Each state is a complex regulatory pattern and can lead the cell to either division or senescence/apoptosis under normal conditions (Harley et al., 1990; d’Adda di Fagagna et al., 2003). Telomere shortening below a critical length results in DNA damage response, end-to-end fusions, and checkpoint-mediated cell cycle arrest or apoptosis (Galati et al., 2013). Here we noted that those embryos originated from HFD oocytes with short telomere displayed high frequency of apoptosis (Figure 5), which may disrupt gametogenesis and adversely impact embryonic/fetal development. An ongoing project is to identify the critical factors mediating the effects of maternal obesity on telomere function. In sum, our findings support a model where maternal obesity induces telomere dysfunction, likely through the disruption of chromosomal stability, consequently contributing to the poor oocyte quality and impaired embryonic developmental potential (Figure 5C).

## Materials and Methods

### Animals and Diet

All animal works and experiments were approved by the Animal Care and Use Committee of Nanjing Medical University. Female ICR mice aged 3 weeks were maintained on a 12/12 h light/dark cycle at constant temperature (22°C) and under specific pathogen-free conditions. Female mice were randomly divided into two groups. For control group, mice were fed with normal diet (D1415, Beijing HFK Bioscience Co., Ltd.) for 16 weeks. For HFD group, mice were fed with high-fat diet containing 35.8% fat, 20.7% protein and 35% carbohydrates (D12492i; Research Diets, Inc.) for 16 weeks.

### Antibodies

Rabbit polyclone anti-TRF1 antibody (Cat#: ab1423) and mouse monoclonal anti-γH2AX antibody (Cat#: ab22551) were purchased from (Cambridge, MA, United Kingdom); Cy5-conjugated goat anti-human IgG was purchased from Jackson Immuno Research Laboratory (West Grove, PA, United States).

### Oocyte Collection, Fertilization, and Embryo Culture

Female mice were superovulated by an intraperitoneal injection of 5 IU pregnant mare serum gonadotropin (PMSG, San Sheng, Ningbo, China). Forty eight hours later, germinal vesicle (GV) stage oocytes were collected from ovary by puncturing the follicles. For *in vivo* MII oocytes, mice received an intraperitoneal injection of 5 IU human Chorionic Gonadotropin (hCG, San Sheng) following with injection of the PMSG 48 h later. Oocytes were retrieved from oviduct ampullae 13.5 h post-hCG, and freed of cumulus cells by exposure to 1 mg/ml hyaluronidase. *In vitro* fertilization was performed according to our previous protocol (Han et al., 2018). Briefly, sperm were isolated from the dissected epididymis of ND ICR mice aged 10–20 weeks and left to capacitate for 1 h in HTF fertilization medium (Millipore, Merck) supplemented with 10 mg/ml BSA. Dispersed spermatozoa were added to HTF drops containing cumulus-oocyte complexes. Then, zygotes were transferred into KSOM medium (Millipore, Merck) and cultured up to blastocyst stage at 37°C in a humidified atmosphere of 5% CO_2_, 5% O_2_, and 90% N_2_.

### Quantitative Real-Time PCR

Pooled oocytes (50 oocytes per sample) were collected for DNA extraction. DNA was extracted using QIAmp DNA micro Kit (Qiagen, Valencia) according to the manufacturer’s instructions. Real-time PCR was performed with SYBR Green using ABI StepOne Plus PCR system (Applied Biosystems). The telomere signal was normalized to the signal from the single-copy gene to generate a T/S ratio indicative of relative telomere length. The related primer sequences are listed in the Supplementary Table 1.

### Telomerase Activity Assay

Telomerase activity was measured by a telomerase PCR ELISA kit (Roche Molecular Biochemicals, Mannheim, Germany) on the basis of manufacturer’s instruction. In brief, telomerase adds telomeric repeats (TTAGGG) to the 3′-end of the biotin-labeled synthetic P1-TS-primer. These elongation products, are amplified by PCR using the primers P1-TS and the anchor-primer P2. After PCR amplification, the products were denatured and hybridized to digoxin-labeled probes that bind to the repeated fragments of the amplified products. The final products are immobilized via the biotin label to a streptavidin fixed on the microporous plate. Immobilized amplicons are detected with the peroxidase labeled anti-diooxin antibody and sensitive peroxidase substrate TMB by the microplate analyzer.

### Immunofluorescence

Immunofluorescence was performed as described previously (Han et al., 2018). Pooled oocytes or embryos were fixed with 3.7% paraformaldehyde for 15 min at room temperature (RT), then permeabilized with 0.5% Triton X-100 for 5 min at RT. After 3 washes in PBS–PVP, they were treated with 1% BSA in PBS for 1 h. Samples were incubated with primary antibody overnight at 4°C. After 3 washes in PBS–PVP, samples were incubated with an appropriate secondary antibody for 1 h at RT. Nuclei was stained with Hoechst 33342 (1:250) for 10 min at RT. To detect cell apoptosis, TUNEL assay (Promega) was performed in accordance with the manufacturer’s instructions. Samples were observed under a laser scanning confocal microscope (LSM 710; Carl Zeiss, Oberkochen, Germany).

### Chromosome Spread

For chromosome spreading in oocytes, oocytes were exposed to Tyrode’s buffer for 30 s at 37°C. Then, samples were fixed in 1% paraformaldehyde and 0.15% Triton X-100 on a glass slide. Oocytes were air-dried and incubated with CREST (1:500). Chromosomes were stained with Hoechst 33342 (Wang et al., 2009). For chromosome spreading in zygotes, cells were incubated in KSOM medium containing 0.1 μg/ml colcemid at 11 h after insemination. Zygotes were exposed to 11% sodium citrate for 20 min at RT and plated onto glass slides, dropping with a freshly methanol: fixative (3:1) mixture of acetic acid. After air drying, chromosomes were stained with Hoechst 33342 (1:250). The laser scanning confocal microscope was used for chromosomes examining.

### Statistical Analysis

Data are presented as means ± SD, unless otherwise stated. All differences were analyzed by Student’s *t*-test using GraphPad Prism 8.0. *P* < 0.05 was considered to be significant.

## Data Availability Statement

The original contributions presented in the study are included in the article/Supplementary Material, further inquiries can be directed to the corresponding author/s.

## Ethics Statement

The animal study was reviewed and approved by the Institutional Animal Care and Use Committee of Nanjing Medical University.

## Author Contributions

JG, CL, HS, and QW designed the research. YX, SZ, YL, ST, LH, and ZH performed the research. JG and QW analyzed the data. JG, HS, and QW wrote the manuscript. All authors contributed to the article and approved the submitted version.

## Conflict of Interest

The authors declare that the research was conducted in the absence of any commercial or financial relationships that could be construed as a potential conflict of interest.
