# Supplementary material for: Telomere Dysfunction in Oocytes and Embryos From Obese Mice
Source: Front Cell Dev Biol. 2021 Jan 21;9:617225. doi: 10.3389/fcell.2021.617225 (PMC7858262; doi:10.3389/fcell.2021.617225)
Supplement: Supplementary file 1 [file Data_Sheet_1.PDF]

Supplemental Table 1

Primer sequences of genes for SiRNA

Gene	***Primer sequence***
Sirt6	F: 5’ –GCAGUGCAUGUUUCGUAUATT– 3’
	R: 5’ –UAUACGAAACAUGCACUGCTT– 3’

Primer sequences of gene for cloning

Gene	***Primer sequence***
Sirt6	F: 5’ –GGCCGGCCATGTGGCAGTCCTCCAGCGTG– 3’
	R: 5’ –GGCGCGCCTCAGCTGGGGGCAGCCTC – 3’

Primer sequences of genes for qRT-PCR

Gene	***Primer sequence***
GAPDH	F: 5’ –CTTTGTCAAGCTCATTTCCTGG – 3’
	R: 5’ –TCTTGCTCAGTGTCCTTGC – 3’
Sirt6	F: 5’ –ATGTCGGTGAATTATGCAGCA– 3’
	R: 5’ –GCTGGAGGACTGCCACATTA– 3’
36B4	F: 5’–ACTGGTCTAGGACCCGAGAAG– 3’
	R: 5’ –TCAATGGTGCCTCTGGAGATT– 3’
Telomere	F: 5’ –CGGTTTGTTTGGGTTTGGGTTTGGGTTTGGGTTTGGGTT– 3’
	R: 5’ –GGCTTGCCTTACCCTTACCCTTACCCTTACCCTTACCCT– 3’
